# Supplementary figures and images for: MGACA-Net: a novel deep learning based multi-scale guided attention and context aggregation for localization of knee anterior cruciate ligament tears region in MRI images
Source: PeerJ Comput Sci. 2023 Jul 13;9:e1483. doi: 10.7717/peerj-cs.1483 (PMC10403161; doi:10.7717/peerj-cs.1483)

Comparison of all models Performance

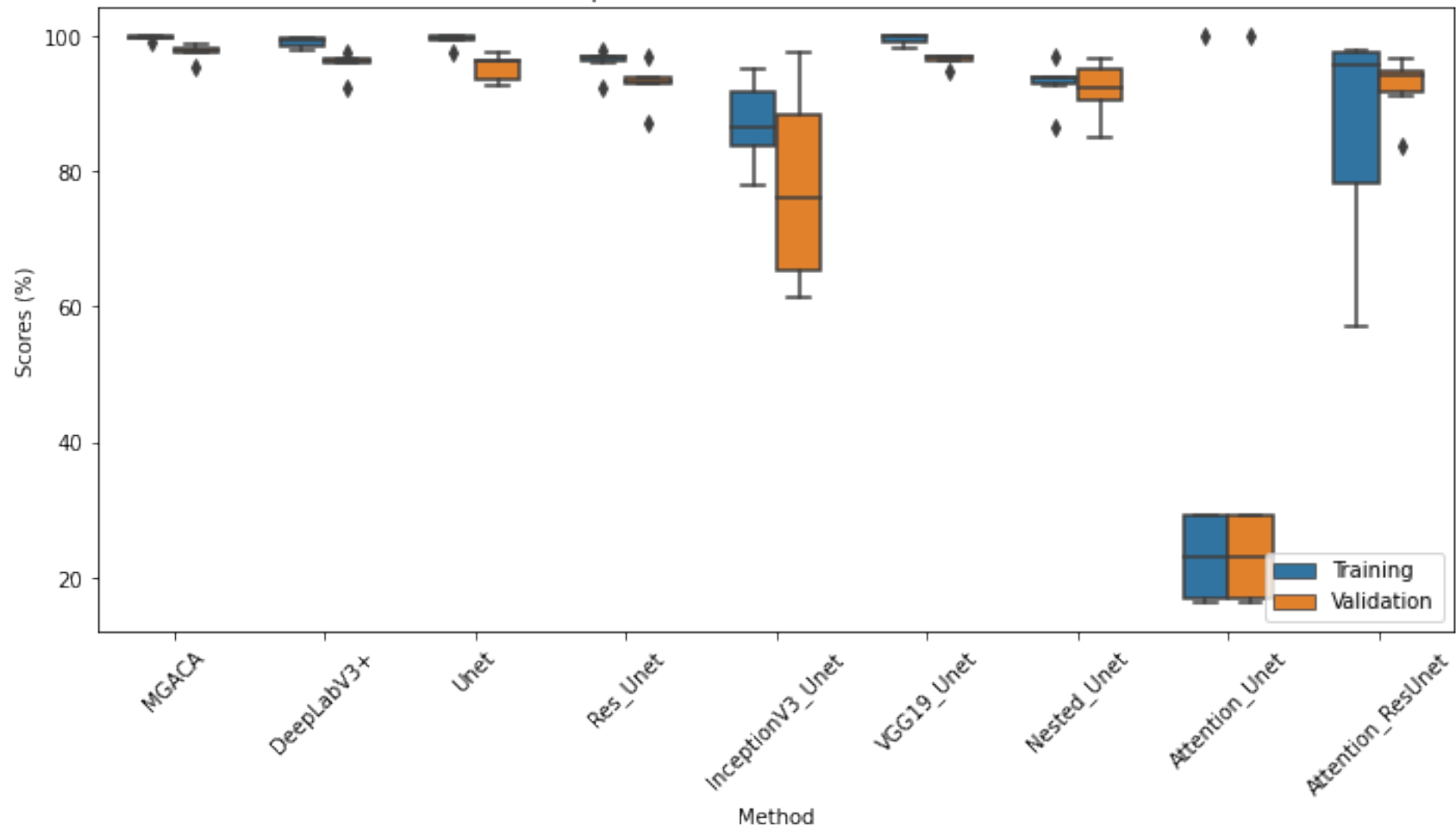

Supplement: Figure S1 [file peerj-cs-09-1483-s003.pdf]

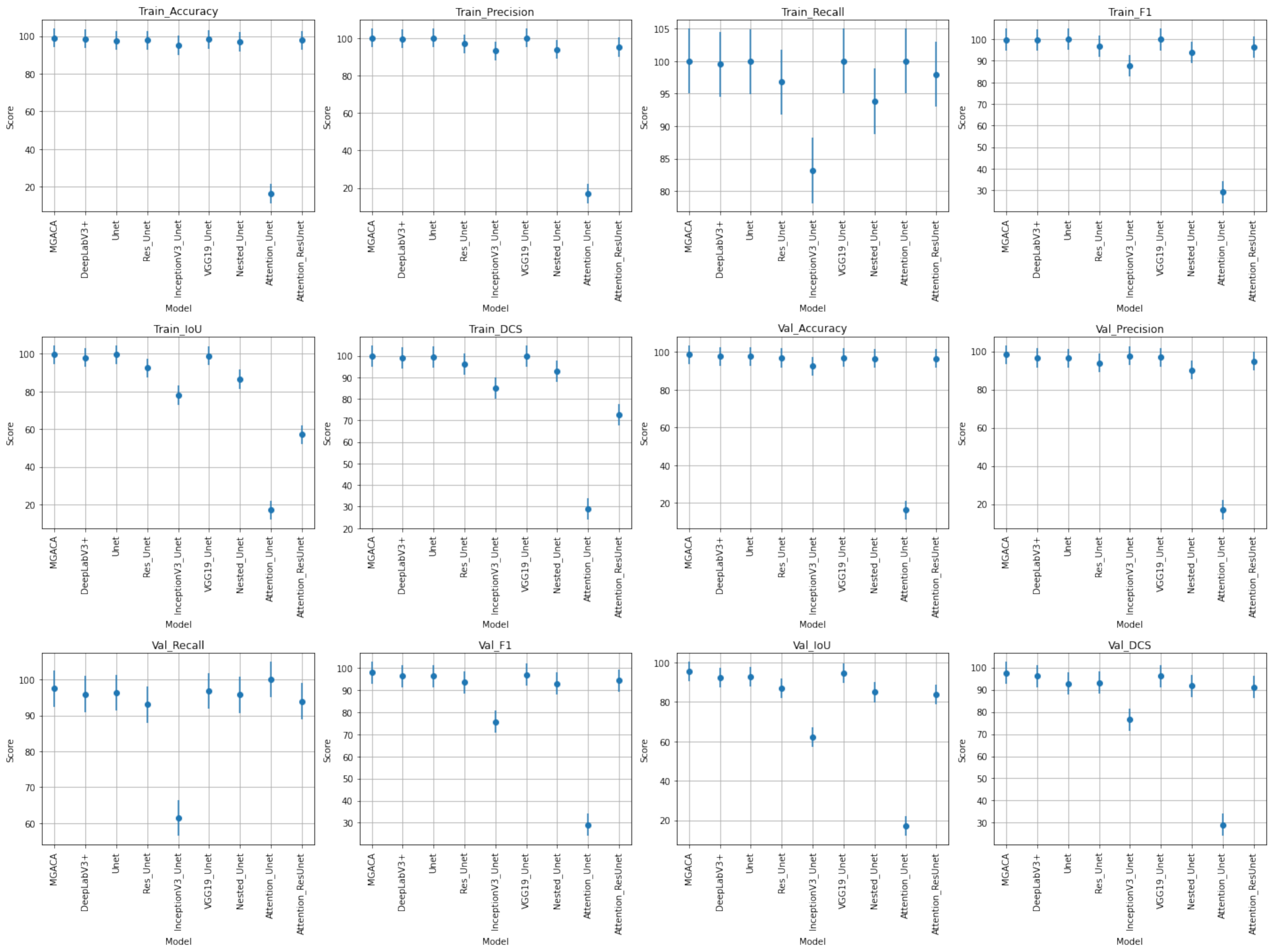

Supplement: Figure S2 [file peerj-cs-09-1483-s004.pdf]

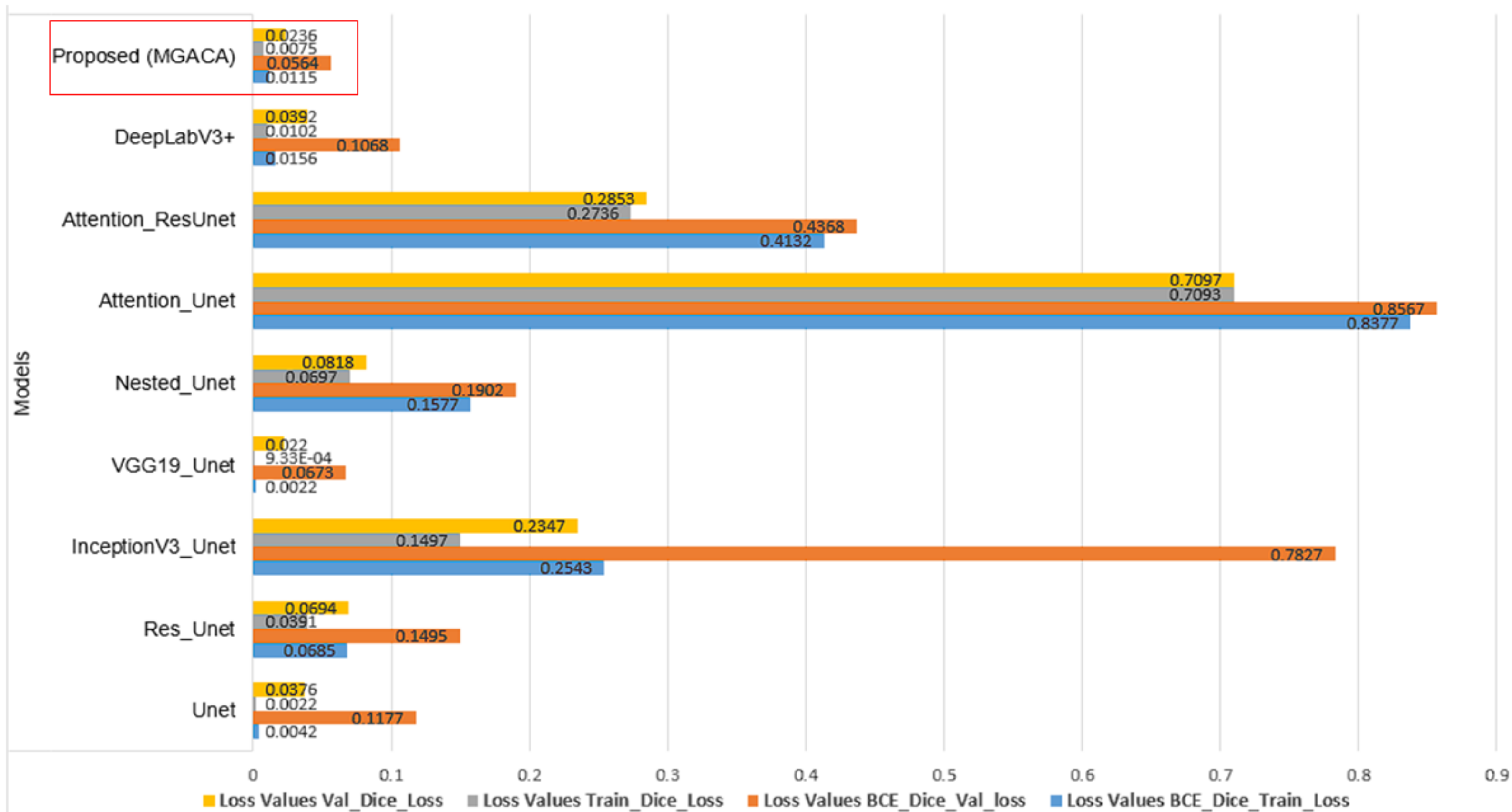

Supplement: Figure S3 [file peerj-cs-09-1483-s005.pdf]
